# Regular Open-Skill Exercise Generally Enhances Attentional Resources Related to Perceptual Processing in Young Males

**DOI:** 10.3389/fpsyg.2020.00941

**Published:** 2020-05-19

**Authors:** Fangyuan Zhou, Xuan Xi, Chaoling Qin

**Affiliations:** ^1^Institute of School Sports Development, Southwest University, Chongqing, China; ^2^Binhai College, Nankai University, Tianjin, China

**Keywords:** open-skill exercise, executive function, ERP, P200, N200

## Abstract

This study aimed to examine whether the regular open-skill exercise led to a selective improvement or a general improvement on cognitive function in healthy young males. Besides, we also intend to expand previous studies by looking into the temporal dynamics of early information processes and cognitive processes through appraising the extensive temporal series of stimulus-locked ERP components. Sixty healthy young males were classified into two groups: those who regularly participated in the open-skill exercise for at least 2 years (*n* = 30), and those who exercised rarely. The participants conducted the Stroop task with event-related brain potential (ERP) recorded. The results indicated that compared with the rare exercise group, the open-skill exercise group led to a selective improvement for accuracy under the incongruent condition. And the open-skill exercise group also led to a selective improvement for response time under the incongruent condition. Moreover, the open-skill exercise group yielded larger P200 amplitudes under both the congruent and incongruent conditions compared with the rare exercise group. The findings suggest that the regular open-skill exercise may promote executive function by an increase in the allocation of attentional resources related to perceptual processing and greater interference control during cognitively demanding tasks in healthy young males.

## Introduction

The benefits of physical activity on cognition function have been continuous observed in lots of studies (Arihiro et al., 2005; Krafft et al., 2014; Gajewski and Falkenstein, 2016), but it appears to be disproportionally distributed. Some studies show that physical activity leads to disproportionately larger modulation of executive function tasks (e.g., incongruent condition during the Stroop task) that require more extensive amounts of cognitive demand (Colcombe and Kramer, 2003; Hillman et al., 2008, 2009; Chang et al., 2014). However, a meta-analysis indicates that physical activity results in a general facilitative modulation of cognitive tasks that involve basic information processing (e.g., congruent and neutral conditions during the Stroop task) (Smith et al., 2010) and executive function (e.g., incongruent condition during the Stroop task). Exercise mode may cause these differences. According to the consistency and predictability of the performing environment, motor skills involved in specific exercise can be divided into open-skill (e.g., soccer, badminton, and volleyball) exercise and closed-skill exercise (e.g., walking and swimming) (Schmidt and Wrisberg, 2008). Compared with the closed-skill exercise, the open-skill exercise requires constant behavioral adaptation in response to unpredictable stimuli and also needs to invest more cognitive resources to increase the efficiency of decision-making processes (Wang et al., 2013). In addition, compared with physical exercise that involves a relatively smaller cognitive requirement, investing more cognitive resources in physical exercise can be more promising benefits to executive function (Huang et al., 2014), so the open-skill exercise may be more beneficial to executive function than closed-skill exercise.

In experimental situations, executive function is often tested by a Stroop task. In the Stroop task, subjects are asked to distinguish the colors in which stimulus words are written. When the word and the color in which it is written are different (e.g., the word “yellow” is written in green), this conflict will result in an increased response time and error rate, and the incongruent condition contains several executive functions such as inhibition, selective attention, and cognitive flexibility (Liotti et al., 2000; Buck et al., 2008). Improvement of task performance may be due to the promotion of processing of relevant information or suppression of the irrelevant stimuli (inhibition) (Neill et al., 1995). The high temporal resolution of ERPs is particularly suitable to investigate the cerebral basis of cognitive function. A visual stimulus contains a series of ERP components. Earlier components (P100, N100, and P200) are related to the early information processes, while later components (N200, P300, and N450) are related to cognitive processes (Rugg and Coles, 1996). An early frontal P200 component which occurs about 150–250 ms is proved to be associated with perceptual processing which requires attention allocation to function (Yuan et al., 2011). Özkaya et al. (2005) found the latency of the P200 component decreased and the amplitude of the P200 component increased in target tones of an auditory oddball paradigm after strength training for 10 weeks in older adults. N200 is frontal-central negativity which occurs about 200–350 ms and is confirmed to relate with the conflict detection process (Chen et al., 2016; Hu et al., 2019). Li et al. (2018) found the open-skill exercisers exhibited smaller N200 amplitudes under both congruent and incongruent Stroop task conditions compared with the rare exercisers in older adults. P300b component which occurs between 300 to 600 ms after stimulus onset is related to the amount of attentional or neural resources allocated to a task (Polich, 2007). Dai et al. (2013) found compared with the rare exercise group, both open-skill and closed-skill exercise groups yielded larger P300b amplitudes under both non-switch and switch conditions of a modified task-switching paradigm in older adults. And Li et al. (2018) found that compared with the rare exercisers, the open-skill exercisers yielded larger P300b amplitude regardless of the Stroop task condition in older adults. N450 occurring approximately 350–500 ms is associated with interference control during the Stroop task (West and Alain, 2000; Imbir et al., 2017). It has been proved that the N450 is related to the selection of competing responses (West and Alain, 1999) or conflict detection (West, 2003). Chang et al. (2017a) found reduced decreased N450 latencies as well as N450 amplitudes across Stroop task conditions following aerobic exercise in young adults.

The existing literature which investigated the relationship between physical activity and cognitive function mostly concentrates on elderly people or adolescents (Stroth et al., 2009; Kirk-Sanchez and McGough, 2013; Zhang et al., 2014). The cognitive function of adolescents is developing and older adults’ cognitive function is declining, their cognitive function is at the stage of alteration, but young adults’ cognitive function is relatively mature and stable. The modulation of cognitive function by physical activity in young adults has received limited attention, and evidence related to modulation of cognitive performance by physical activity is less clear and sparse. A meta-analysis indicated that physical exercise showed greater modulation of cognitive performance in adolescents and older adults than young adults (Chang et al., 2012). In most studies, the duration of physical activity lasted more than 3 months (Chang et al., 2017b; Tsai et al., 2017). But physical exercise showed smaller modulation in young adults, so this study prolongs the duration of exercise. In general, the purpose of this study is to examine whether the open-skill exercise for at least 2 years leads to a selective improvement or a general improvement on cognitive function in healthy young males. Besides, we intend to expand previous studies by looking into the temporal dynamics of early information processes and cognitive processes through appraising the extensive temporal series of stimulus-locked ERP components. The current study involved a cross-sectional design to find the difference in cognitive function between the open-skill and rare exercise groups. Based on previous studies in older adults (Dai et al., 2013; Chang et al., 2017b; Li et al., 2017), we hypothesized that compared with the rare exercise group, the open-skill exercise group would exhibit higher accuracy and faster response time during the Stroop task. Moreover, we predicted that the open-skill exercise group would yield larger P300b amplitude(s) than the rare exercise group during the Stroop task.

## Materials and Methods

### Participants

Sixty male college students were recruited via advertisements from Southwest University in China. All participants were required to meet the following criteria: between the ages of 18 and 28 years, right-handed, normal or corrected-to-normal vision as well as color perception, the body mass index (BMI) less than 28, and no history of psychiatric, cardiovascular disease, neurological disorders, or physical disability. Moreover, all participants must pass an exercise screening. Participants in the open-skill exercise group (*n* = 30) took the open-skill exercise (i.e., table tennis, tennis, badminton, basketball, soccer, and volleyball) at least three times a week for more than an hour per session with at least a moderate level of intensity (heart rate > 110 times/min) in the previous 2 years (Dai et al., 2013). While participants in the rare exercise group (*n* = 30) did exercise no more than two times a week for less than 30 min per session as well as at the lower level of intensity in the previous 2 years (Dai et al., 2013). The rare exercise wasn’t a type of exercise, it meant participants seldom did exercise. In daily life, participants had to walk, had physical education class, etc., so we couldn’t require them not to do exercise at all. Before the experiment, participants were required to fill in the Edinburgh handedness inventory (Oldfield, 1971), the international physical activity questionnaire (IPAQ) (Hagströmer et al., 2006) the physical activity readiness questionnaire (PAR-Q) (American College of Sports Medicine, 2006) as well as the shortened version of Raven’s Advanced Progressive Matrices test (sRAPM) (Marzecová et al., 2013). Participants’ demographics and physical characteristics are summarized in Table 1. The ethics committee of Southwest University approved this experiment, and the informed consent forms were signed by all participants before this experiment. This experiment was compliant with the ethical principles of Declaration of Helsinki (World Medical Organization, 1996).

**TABLE 1:** Data for participants’ demographic and physical characteristics between two groups (mean ± SE).

	Open-skill exercise (*n* = 30)	Rare exercise group (*n* = 30)
Age (year)	21.00 ± 0.38	20.43 ± 0.27
Height (cm)	174.87 ± 1.11	173.8 ± 0.92
Weight (kg)	66.47 ± 1.21	63.30 ± 1.07
BMI (kg/m^2^)	21.69 ± 0.25	20.95 ± 0.30
IQ^*a*^	11.94 ± 0.24	12.62 ± 0.26
Exercise experience		
Tennis	13	–
Basketball	6	–
Badminton	4	–
Table tennis	3	–
Soccer	2	–
Volleyball	2	–
Years (regular)	3.50 ± 0.27	0.33 ± 0.07
Duration/session (min)	99.33 ± 9.18	23.17 ± 2.11
Frequency/week	4.47 ± 0.19	1.80 ± 0.12
IPAQ (METs)	5783.80 ± 598.06	1537.23 ± 147.39

*BMI, body mass index; IPAQ, International physical activity questionnaire; METs, metabolic equivalents. ^*a*^As measured by a shortened version of Raven’s Advanced Progressive Matrices test (sRAPM).*

### Stroop Task and Experimental Procedure

The Stroop task used only one block which consisted of two types of stimuli conditions: congruent and incongruent. 60 incongruent trials consisted of four color words printed in Chinese [i.e., 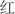 (RED), 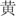 (YELLOW), 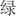 (GREEN), or 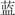 (BLUE)], each printed in a color not matching the word meaning [e.g., 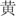 (YELLOW) printed in green ink color]. While 60 congruent trials consisted of the four words printed in the color matching the word meaning (e.g., 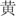 (YELLOW) printed in yellow ink color). The presentation of the two types of stimuli was randomized.

**FIGURE 1:**
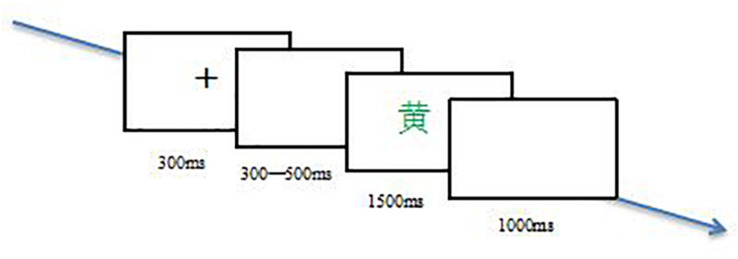
Schematic illustration of the behavioral procedure in a trial.

Prior to the experiment, participants were told they would take part in a keystroke task. In an aurally isolated room, participants sat about 80 cm away from a computer screen. Participants were required to put their left middle, left index, right index as well as right middle fingers on the s, d, j, as well as k keys, each of them represented a color. Each trial started with the 300 ms onset of a white, fixation cross in the center of a black display screen. And then between 300 and 500 ms following the disappearance of the cross, a Chinese word which was printed in one of four colors presented. Participants were asked to identify the color in which the word was printed as accurately and quickly as possible by pressing the key within 1500 ms. A 1000 ms blank screen presented following each Chinese word (Figure 1). A practice session consisting of 10 trials repeated until an accuracy rate reached 90%, and then participants could take part in the experiment session. In the whole practice and experiment sessions, participants were required to focus on the computer screen.

### ERP Recording and Analysis

Electroencephalographic (EEG) was recorded using an elastic cap with 32 scalp sites placed according to the International 10–20 system (Neuroscan). A ground electrode was placed on Fpz, and reference electrodes were located on the left and right mastoids. The EEG was amplified using a DC-100 Hz bandpass, with digitizing at a sample rate of 250 Hz. All impedance of electrodes was maintained below 10 KΩ.

Event-related brain potential data were analyzed through the EEGLAB software (Delorme and Makeig, 2004). EEG data were filtered using 0.1 Hz high-pass as well as 30 Hz low-pass zero-phase filters. EEG data were extracted from 200 ms pre-stimulus onset to 1000 ms post-stimulus onset, and the 200 ms pre-stimulus period was used to baseline correct. Run Independent Component Correlation Algorithm (runICA) was used to correct eye movement artifacts (blinks and eye movements) (Delorme and Makeig, 2004). Trials with response errors as well as artifacts exceeding ±100 μV were also excluded. ERP data averages were computed for each participant for each condition, separately. Based on previous studies (Yuan et al., 2011; Chang et al., 2017a; Ligeza et al., 2018) as well as the grand-averaged ERP waveforms, the P2 component occurred about 170–210 ms at fronto-central scalp sites (i.e., Fz electrodes), the N2 component occurred about 260–330 ms at fronto-central scalp sites (i.e., Fz electrodes), the P3b component occurred about 360–420 ms at the centro-parietal scalp sites (i.e., Pz electrodes), and the N450 component occurred about 430–490 ms at fronto-central scalp sites (i.e., Fz electrodes). Therefore, the P2, N2, P3b, and N450 components were measured as the difference waves between the incongruent and the congruent conditions at the specified time windows as well as electrodes. Besides, the topographic scalp distributions of the specific components which were illustrated according to all 32 electrode sites with spherical interpolation were presented.

### Statistical Analysis

Data were analyzed in SPSS v. 17 (SPSS Inc., Chicago, IL, United States). The discrepancies of participants’ characteristics between open-skill and rare exercise groups were examined through an independent-sample *T* Test.

A 2 (Groups: open-skill exercise group vs. rare exercise group) × 2 (Tasks: congruent vs. incongruent) repeated measures ANOVA was used for accuracy and response time, individually. As to ERP amplitude and latency, a 2 (Groups) × 2 (Tasks) repeated measures ANOVA was conducted for the P2 component (at Fz in the 170–210 ms time window), the N2 component (at Fz in the 260–330 ms time window), the P3b component (at Pz in the 360–420 ms time window), and the N450 component (at Fz in the 430–490 ms time window), individually. A Greenhouse-Geisser correction was used to adjust for family-wise error when the assumption of sphericity was violated. *Post hoc* comparisons were conducted through Bonferroni significant difference tests. The family-wise alpha level was set at 0.05.

## Results

### Participant Characteristics

An independent-sample *T* Test revealed non-significant in age, height, weight, BMI, and IQ between the two group, *t*(1,58) = 1.22, 0.74, 1.96, 1.90, 1.96, *p*s > 0.05. As to physical characteristics, the participants in the rare exercise group had less exercise experience (i.e., years of regular exercise, duration per session, and frequency per week) [*t*(1,58) = 11.52, 8.08, 11.82, *p*s < 0.001] and lower levels of physical activity (METs) [*t*(1,58) = 6.89, *p* < 0.001] than those in the open-skill exercise group.

### Behavioral Data

Table 2 presents the accuracy and response time for each condition.

**TABLE 2:** Behavioral data across Stroop task conditions between two groups (mean ± SE).

Behavioral data	Open-skill exercise group		Rare exercise group	
	Congruent	Incongruent	Congruent	Incongruent
Accuracy (% ± SE)	96.89 ± 0.59	93.78 ± 0.69	97.28 ± 0.50	90.83 ± 0.99
RT(ms ± SE)	672.26 ± 14.66	770.70 ± 17.92	692.69 ± 11.95	828.33 ± 16.33

#### Accuracy

The repeated measures ANOVA showed a significant main effect for task, *F*(1,58) = 77.52, *p* < 0.001, partial η^2^ = 0.57, with the incongruent task (92.31 ± 0.63%) yielding lower accuracy than the congruent task (97.08 ± 0.38%). There was a significant interaction between group × task, *F*(1,58) = 9.43, *p* = 0.003, partial η^2^ = 0.14. Following analyses showed that the open-skill (96.89 ± 0.59%) and rare exercise (97.28 ± 0.50%) groups didn’t show significant difference in the congruent task, but the open-skill exercise group (93.78 ± 0.69%) yielded higher accuracy than the rare exercise group (90.83 ± 0.99%) in the incongruent task, *p* = 0.018, and the analyses also showed that the incongruent task yielded lower accuracy than the congruent task in both groups, *p*s < 0.001. No significant main effect for group was found.

#### Response Time

The repeated measures ANOVA showed a significant main effect for task, *F*(1,58) = 414.75, *p* < 0.001, partial η^2^ = 0.88, with the congruent task (682.48 ± 9.47 ms) yielding shorter response time than the incongruent task (799.52 ± 12.59 ms). There was a significant interaction between group × task, *F*(1,58) = 10.47, *p* = 0.002, partial η^2^ = 0.15. Following analyses showed that the open-skill (672.26 ± 14.66 ms) and rare exercise (692.69 ± 11.95 ms) groups didn’t show significant difference in the congruent task, but the open-skill group (770.70 ± 17.92 ms) yielded shorter response time than the rare exercise group (828.33 ± 16.33 ms) in the incongruent task, *p* = 0.021, and the analyses also revealed that the congruent task yielded shorter response time than the incongruent task in both groups, *p*s < 0.001. No significant main effect for group was found.

#### ERP Data

**FIGURE 2:**
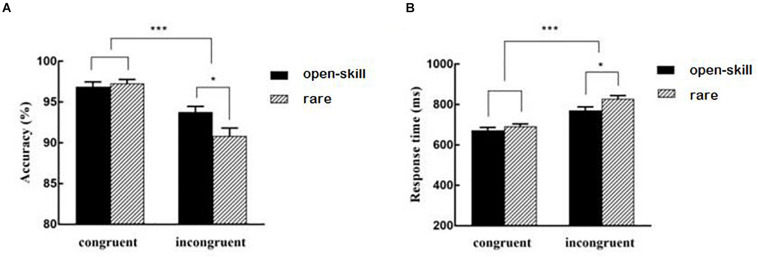
The interaction effects between group × task **(A)** accuracy **(B)** response time. *p < 0.01, ***p < 0.001.

**FIGURE 3:**
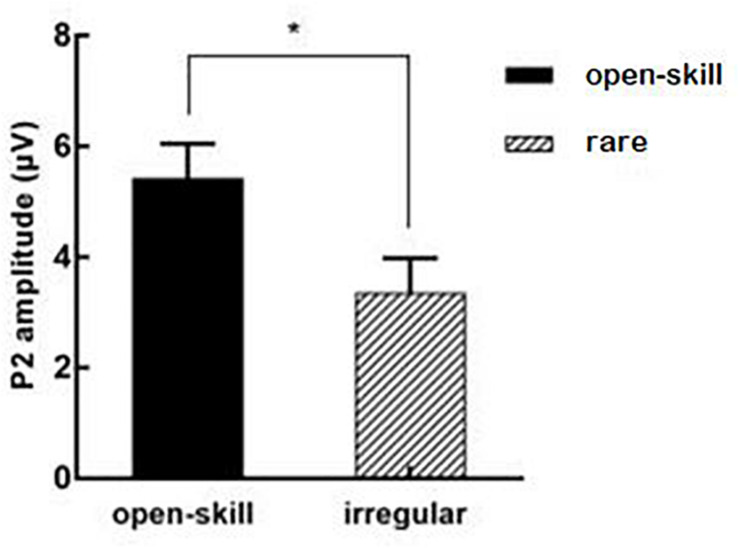
The main effect of P2 amplitude for group. *p < 0.05.

**FIGURE 4:**
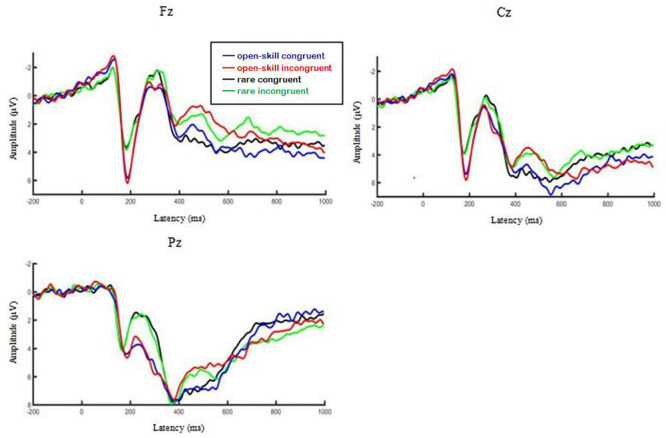
The grand-averaged ERP waveform for each condition between two groups.

**FIGURE 5:**
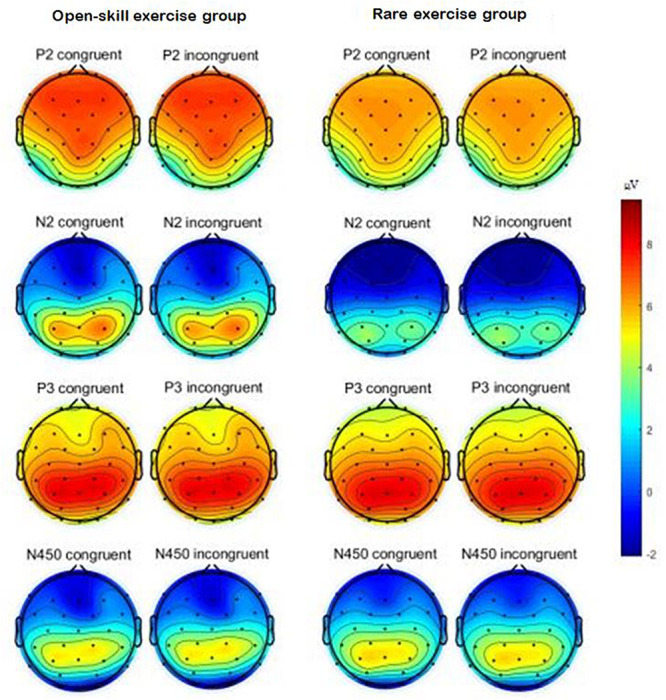
The topographic scalp distribution of P2, N2, P3, and N450 components between two groups.

Table 3 presents ERP values for each condition. Figure 2 illustrates the interaction effects between group × task (a) accuracy (b) response time. Figure 3 presents the main effect of P200 amplitude for group. Figure 4 presents the grand-averaged ERP waveform for each condition between two groups, and Figure 5 illustrates the topographic scalp distributions of the P200, N200, P300, and N450 components between two groups.

**TABLE 3:** ERP data for each Stroop task condition between two groups (mean ± SE).

Component	Open-skill exercise group		Rare exercise group	
	Congruent	Incongruent	Congruent	Incongruent
Amplitude (μV)				
P2	5.30 ± 0.64	5.57 ± 0.62	3.31 ± 0.59	3.42 ± 0.71
N2	−0.50 ± 0.74	−0.68 ± 0.72	−1.41 ± 0.57	−1.42 ± 0.45
P3	7.59 ± 0.74	7.35 ± 0.79	7.67 ± 0.72	7.63 ± 0.71
N450	2.26 ± 0.69	0.84 ± 0.51	3.14 ± 0.73	1.51 ± 0.70

Latency (ms)				

P2	192.27 ± 3.28	191.60 ± 3.27	187.47 ± 4.17	191.33 ± 4.15
N2	296.13 ± 6.34	299.33 ± 6.24	290.93 ± 5.73	300.40 ± 6.67
P3	381.20 ± 4.71	378.27 ± 4.29	387.33 ± 4.95	385.87 ± 4.59
N450	448.67 ± 5.57	460.67 ± 5.92	454.80 ± 6.29	463.07 ± 5.71

#### P200 Component

In terms of amplitude, the repeated measures ANOVA showed a significant main effect for group, *F*(1,58) = 5.59, *p* = 0.021, partial η^2^ = 0.09, with the open-skill exercise group (5.43 ± 0.62 μV) yielding larger amplitude than the rare exercise group (3.36 ± 0.62 μV). No significant main effect for task or two-way interaction was found.

As to P200 latency, no significant main effects or two-way interaction were found.

#### N200 Component

In terms of amplitude, no significant main effects or two-way interaction were observed.

As to N200 latency, no significant main effects or two-way interaction were found.

#### P300 Component

In terms of amplitude, no significant main effects or two-way interaction were observed.

As to P300 latency, no significant main effects or two-way interaction were found.

#### N450 Component

In terms of amplitude, the analyses showed a significant main effect for task, *F*(1,58) = 27.65, *p* < 0.001, partial η^2^ = 0.32, with the congruent task (2.70 ± 0.50 μV) yielding less negative amplitude than the incongruent task (1.17 ± 0.43 μV). No significant main effect for group or two-way interaction was found.

As to N450 latency, no significant main effects or two-way interaction were found.

## Discussion

This study intended to investigate the open-skill exercise for at least 2 years led to a selective improvement or a general improvement on cognitive function in healthy young males. In addition, the purpose of this study was also to expand previous research by investigating multiple ERP components (e.g., P200, N200, P300b, and N450). The present study found that compared with the rare exercise group, the open-skill exercise group led to a selective improvement for accuracy as well as response time under the incongruent task condition. Moreover, the open-skill exercise group generally yielded larger P200 amplitudes under both congruent and incongruent conditions compared with the rare exercise group.

### Task Performance

The study found that compared to the incongruent condition, the accuracy of the congruent condition was higher and the response time was shorter, which suggested that the congruent condition needed a lower amount of inhibitory control than incongruent condition (Chang et al., 2014). This finding not only reflected the typical “Stroop effect” (Ridley Stroop, 1992) but also replicated previous researches (Chang et al., 2015, 2017a; Li et al., 2017), and demonstrated the appropriateness of our task manipulation. Compared to the rare exercise group, the open-skill exercise group led to a selective improvement for accuracy under the incongruent task condition which replicated previous findings in older adults (Chang et al., 2017b). As to response time, the open-skill exercise group also led to a selective improvement under the incongruent task condition than the rare exercise group which replicated previous findings in older adults (Chang et al., 2014). These results not only demonstrated that the facilitation in cognitive performance wasn’t the result of a speed-accuracy trade-off but also demonstrated that the open-skill exercise selectively improves task which necessitates greater amounts of inhibitory control.

### P200 Component

Compared with the rare exercise group, larger P200 amplitude was observed for the incongruent condition in the open-skill exercise group. Although the N200 and P300 components have been the primary emphasis in the response inhibition researches, some earlier components (e.g., P200) may also put forward an important influence in the process of inhibition success (Thomas et al., 2009; Benikos et al., 2013). The earlier frontal P200 component has been related to an index of the intensity of perceptual processing which needs attention allocation to function (Chen et al., 2007; Yuan et al., 2011). These findings might suggest that in order to protect against interference from irrelevant stimuli, the open-skill exercise mobilized more attentional resources (Garcia-Larrea et al., 1992), which gave a clear path to the imperative stimulus for further processing (Oades, 1998). Enhanced P200 amplitude was related to concurrent reductions in reaction time and commission errors (Johnstone et al., 2005). In line with this, this study found the open-skill exercise group yielded shorter response time and higher accuracy for the incongruent task than the rare exercise group. However, larger P200 amplitude was also found in the congruent task, which indicated the open-skill exercisers might also engage more attentional resources associated with basic information processing than the rare exercisers. Collectively, our study found the open-skill exercise might generally enhance attentional resources related to perceptual processing, regardless of Stroop task types.

### Other ERP Components

We also examined several other waveform ERP components. For instance, smaller N450 amplitude was observed for congruent task than incongruent task in the frontal-central region which replicated findings from previous studies (Chang et al., 2017a; Zhou and Qin, 2019). N450 is sensitive to conflict monitoring processes (Badzakova-Trajkov et al., 2009; Larson et al., 2014). And the N450 component has been proved to reflect activation of the anterior cingulate cortex (Liotti et al., 2000) which was related to the selection of competing responses (West and Alain, 1999) or conflict detection (West, 2003). So the larger N450 amplitude for incongruent condition might reflect monitoring processes which were involved in conflict detection. Regarding the N200 component, the open-skill exercise didn’t showed modulation of the N200 component. However, Li et al. (2018) found compared to the rare exercise group, the open-skill exercise group yielded smaller N200 amplitudes during the Stroop task in older adults. As to the P300b component, this study didn’t find the significant difference of the P300b component between open-skill exercisers and rare exercisers, either. Previous studies usually reported physical exercise was associated with larger P300b amplitude in older adults (Dai et al., 2013; Chang et al., 2017b; Tsai et al., 2017). A meta-analysis suggested that compared to young adults, physical exercise showed greater modulation of cognitive performance in adolescents and older adults (Chang et al., 2012). So the possible explanation about the N2 and P3 components might be the population we selected. Unlike the cognitive function of adolescents is developing or older adults’ cognitive function is declining, young adults’ cognitive function is relatively mature and stable, so physical exercise showed smaller modulation.

### Limitations

There are some limitations to our study. First, our study used a cross-sectional design to whether the open-skill exercise for at least 2 years led to a selective improvement or a general improvement on cognitive function in healthy young males. However, this kind of experimental design can only explore the correlation instead of the causality. Second, this study only included young males, so we can’t ensure these findings can expand to young females or other populations. Last, this study didn’t include the closed-skill group, so we couldn’t investigate the different modulation of cognitive domains as well as neural processes by the two exercises modes.

## Conclusion

It is concluded that the regular open-skill exercise may promote executive function by an increase in the allocation of attentional resources related to perceptual processing and greater interference control during cognitively demanding tasks in healthy young males.

## Data Availability Statement

The datasets generated for this study are available on request to the corresponding author.

## Ethics Statement

The studies involving human participants were reviewed and approved by the ethics committee of Southwest University. The patients/participants provided their written informed consent to participate in this study.

## Author Contributions

All authors listed have made a substantial, direct and intellectual contribution to the work, and approved it for publication.

## Conflict of Interest

The authors declare that the research was conducted in the absence of any commercial or financial relationships that could be construed as a potential conflict of interest.
